# Machine Learning Modeling of Wine Sensory Profiles and Color of Vertical Vintages of Pinot Noir Based on Chemical Fingerprinting, Weather and Management Data

**DOI:** 10.3390/s20133618

**Published:** 2020-06-27

**Authors:** Sigfredo Fuentes, Damir D. Torrico, Eden Tongson, Claudia Gonzalez Viejo

**Affiliations:** 1 Digital Agriculture, Food and Wine Sciences Group, School of Agriculture and Food, Faculty of Veterinary and Agricultural Sciences, University of Melbourne, Melbourne, VIC 3010, Australia; eden.tongson@unimelb.edu.au (E.T.); cgonzalez2@unimelb.edu.au (C.G.V.); 2 Department of Wine, Food and Molecular Biosciences, Faculty of Agriculture and Life Sciences, Lincoln University, Lincoln 7647, New Zealand; damir.torrico@lincoln.ac.nz

**Keywords:** sensory profile, chemical fingerprinting, water balance, artificial intelligence, wine color

## Abstract

Important wine quality traits such as sensory profile and color are the product of complex interactions between the soil, grapevine, the environment, management, and winemaking practices. Artificial intelligence (AI) and specifically machine learning (ML) could offer powerful tools to assess these complex interactions and their patterns through seasons to predict quality traits to winegrowers close to harvest and before winemaking. This study considered nine vintages (2008–2016) using near-infrared spectroscopy (NIR) of wines and corresponding weather and management information as inputs for artificial neural network (ANN) modeling of sensory profiles (Models 1 and 2 respectively). Furthermore, weather and management data were used as inputs to predict the color of wines (Model 3). Results showed high accuracy in the prediction of sensory profiles of vertical wine vintages using NIR (Model 1; R = 0.92; slope = 0.85), while better models were obtained using weather/management data for the prediction of sensory profiles (Model 2; R = 0.98; slope = 0.93) and wine color (Model 3; R = 0.99; slope = 0.98). For all models, there was no indication of overfitting as per ANN specific tests. These models may be used as powerful tools to winegrowers and winemakers close to harvest and before the winemaking process to maintain a determined wine style with high quality and acceptability by consumers.

## 1. Introduction

The viticulture and winemaking industries have been accumulating important data from past vintages for record-keeping, related mainly to operations and management practices, such as machinery usage, fertilization, irrigation scheduling pest, and disease incidence, and control applications [1]. Other wineries keep records of physicochemical characteristics and/or sensory profiles related to berry and wine quality traits, either done at chemistry laboratories or in-house, with some of these vineyards with records of more than 15 growing seasons. Keeping with digital technological advances, these management tools can be found in the form of computer, smartphone, and tablet PC applications for portability and easy access to records [2]. However, there have been minimal attempts to analyze these records using new and emerging tools, such as data mining and machine learning. Most new researches have been focused on the implementation of robotic platforms and unmanned aerial and terrestrial vehicles to acquire remote sensing data to obtain information for decision-making related to irrigation scheduling, pest and disease detection or yield estimation, among others [3,4,5,6,7].

Specifically, research taping into records using machine learning has been recently applied to a robotic dairy farm, analyzing and modeling four years of data using machine learning to assess milk quality traits and productivity [8]. Machine learning modeling was implemented in a vineyard from vertical vintages and meteorological data to obtain aroma profiles according to changes in seasonality [9]. The latter showing that quality trait aspects from wines produced can be characterized and modeled.

Berries and wine sensory profiles, such as color, anthocyanin content, aroma profiles, astringency, and mouthfeel, among others, are dependent on berry quality traits [10,11] as a product of the grapevine and soil interaction [12,13], management practices, and seasonal conditions [14,15]. Further aroma profiles are expressed as product of selection of yeasts [16,17,18], the winemaking technique [19], and wine aging [20,21].

The effects of management practices such as canopy management [22,23], specifically in terms of pruning techniques [23,24], canopy training systems [23,24,25], fertilization management [26,27,28,29,30], and irrigation scheduling, on berry and wine quality traits have been widely reported. Irrigation scheduling has been recognized as one of the main management practices to manipulate berry size and compounds in berries, specifically by using two techniques, namely regulated deficit irrigation (RDI) [31,32,33,34] and partial rootzone drying (PRD) [35,36]. These techniques are mostly applied from veraison to harvest (V-H) since the maximum benefit can be achieved for berry quality traits, such as sugar content, skin to flesh ratio, anthocyanin, and polyphenol content, and with minimal impact on yield.

Seasonality has a natural variability. However, this variability has been exacerbated in the past 20 years due to climate change [37,38] affecting wine quality traits through droughts, excessive rain, increased ambient temperatures, frosts, heatwaves, and bushfires. It can be said that the last half of the 20th century was benefited by climate change conditions (i.e., increased ambient temperatures with less climatic anomalies), which expressed most of the cultivar characteristics from different wine regions around the world [39,40]. From the first 20 years in the 21st century, higher ambient temperatures and climatic anomalies have resulted in severe droughts and changes in key phenological stages of grapevines, affecting productivity and quality of grapes [41,42]. One major consequence is the dual warming impact, wherein the increase in temperature due to climate change causes the compression of phenological stages and earlier maturity of grapes and results in early harvest during the warmer months producing excessive atmospheric demands [43,44]. This could force grapevines to extract water from wherever possible, even from berries producing berry shriveling and patterns of cell death within the mesocarp of berries [45,46,47]. Berry cell death has shown to be directly linked with berry quality and aroma profiles [48]. Specifically, higher temperatures imposed on Shiraz and Chardonnay treatments in the vineyards resulted in increased cell death patterns and rates.

Machine learning (ML), which is part of artificial intelligence (AI) is a powerful predictive tool that can be used to analyze and model complex processes such as winegrowing and winemaking. ML and other AI tools have been previously applied for different beverages such as beer, sparkling water, cider, and wines to assess their quality and consumer preferences after the beverage has been produced [49]. For Pinot Noir, recent research has shown the applicability of ML modeling using weather and management practices as inputs to model the aroma profile of resulting wines in a vertical vintage assessment [50]. This research offers an integrative ML tool based on near-infrared spectroscopy (NIR) from wines from a vertical vintage (Model 1) and the effects of seasonal weather patterns and water management practices (Model 2) to assess sensory profiles of wines before the winemaking process. Furthermore, weather data and management practices were used to predict wine color in three different color-scales (i) CIELab, (ii) RGB, and (iii) CMYK (Model 3). This information can be used by winemakers to adjust the process to obtain more consistent wine styles, which can be recognized by consumers.

## 2. Materials and Methods

### 2.1. Vineyards and Samples Description

Data from nine different vintages (2008–2016) of Pinot Noir wine were obtained from a boutique vineyard (14.5 ha) located in the South of the Great Dividing Range of the Macedon Ranges in Romsey/Lancefield, Victoria in Australia at an elevation of 540 m.a.s.l. The vineyard is located in a region with fresh and cool evenings starting in the autumn season, which allows slow ripening as well as maintenance of the natural acidity. Being a commercial boutique vineyard, Pinot Noir wines are produced under controlled processing methods and using only berries from the site and fermented using wild yeast from the vineyard/winery. These were then matured in French oak for 20–22 months. Samples of each vintage (wine bottles 750 mL) were obtained from the online store of the vineyard (Table 1).

### 2.2. Weather Data Acquisition

As described in the publication from Fuentes et al. [50], integrative weather information for each vintage was obtained from the Bureau of Meteorology (BoM). The derived weather parameters based on temperature and rainfall data consisted of (i) degree days from September to harvest (DD-S-H), (ii) maximum January temperature (MJT), (iii) mean maximum temperature from veraison to harvest (MeanMaxTV-H), (iv) mean minimum temperature from veraison to harvest (MeanMinTV-H), and (v) water balance (WB).

For degree days, which is considered as a thermal time, it was calculated with base 10 °C from hourly temperature data reconstructed from readily available maximum and minimum daily temperature data from BoM. The hourly temperature (T_H_) reconstruction was obtained using the method proposed by Zhang et al. (2016) [51] and using the following formula:

(1) $$DD=\overset{23:59}{\underset{00:00}{\sum }}T_{H}>10\;{}^{\circ}C$$

where DD = degree days; T_H_ = hourly temperature in °C.

Water balance (WB) was calculated based on values of irrigation in megaliters (I; ML), effective rainfall (RF), and evapotranspiration (ET_c_) calculated using the corresponding crop coefficient (Kc) for different phenological stages. Specific crop coefficients (Kc) used were based on those considered by Collins et al. [52]. The 0.85 fraction corresponds to effective rain, which can be readily available to plants [53]:

(2) $$WB=I+RF\left(0.85\right)-ET_{c}$$

### 2.3. Near-Infrared Spectroscopy and Color Data Analysis

Triplicates from two bottles of each of the wine samples were analyzed three times each (*n* = 9 readings) using a near infra-red (NIR) spectroscopy handheld device (microPHAZIR™ RX Analyzer; Thermo Fisher Scientific, Waltham, MA, USA). This machine is capable of acquiring the spectra within the 1596–2396 nm range with readings every 7–9 nm. Whatman^®^ qualitative grade three filter paper of 7 cm (Whatman plc., Maidstone, UK) was saturated with the wine samples and then read with the device. The NIR values of the filter paper were subtracted to obtain the values for wine. This method was described and validated in a study by Gonzalez Viejo et al. [54].

Color was measured in triplicates using the NIX™ PRO color sensor (Nix Sensor Ltd., Ontario, Canada) with D50 illuminant and 10° observer. A total of 15 mL of wine was poured into a 35 mm × 10 mm Corning^®^ CellBIND^®^ Petri dish (Sigma -Aldrich Inc., St. Louis, MO, USA) and placed over a generic/unbranded Light Emitting Diode (LED) light pad (Hong Kong) and measured with the NIX™ PRO device connected via Bluetooth to a smartphone and the NIX™ PRO color sensor application (App). Data were obtained in three color scales (i) CIELab, (ii) Red, Green, Blue (RGB), and (iii) Cyan, Magenta, Yellow, Black (CMYK).

### 2.4. Descriptive Sensory Evaluation

A sensory panel of 12 participants from The University of Melbourne (Ethics ID: 1545786.2) was trained using a combination of International Standard methodology (ISO 8586-1: 1993E Sensory analysis–General guidelines for the selection, training, and monitoring of selected assessors and expert sensory assessors, and quality control procedures) [55] and the quantitative descriptive analysis method (QDA^®^). The training details are described in the study published by Gonzalez Viejo et al. [56], using panelists that were regular wine consumers and with training designed using wine samples and references related to red wine. Once the panelists were trained, a blind sensory session was conducted in the sensory laboratory at The University of Melbourne, which consists of individual booths with uniform lighting. The number of samples (N = 9) was adequate and enough for sensory evaluation to avoid fatigue of the panelists due to the alcohol concentration and astringency present in the wine, this makes the results more reliable. This is in accordance with the recommended maximum number of samples, which is usually between six and twelve for descriptive sensory evaluation [57,58].

The sensory questionnaire was displayed in Android (Google, Mountain View, CA, USA) Tablets using the Bio-Sensory App (The University of Melbourne, Parkville, Vic, Australia) [59]. Table 2 shows the descriptors assessed by the panelists, which were rated using a 15-cm non-structured scale. Samples were served at 20 °C in International Standard Wine Tasting Glasses by Luigi Bormioli, and the serving size was 30 mL.

### 2.5. Statistical Analysis and Machine Learning Modeling

An analysis of variance (ANOVA) was performed for the sensory and color data to evaluate significant differences between samples for each parameter. Fisher’s least significant difference (LSD) *post hoc* test was conducted for pairwise comparisons using α = 0.05.

**Figure 1:**
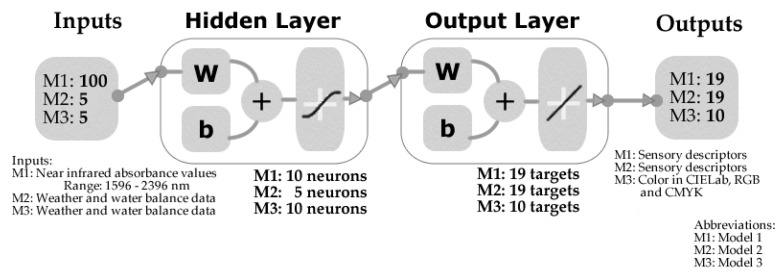
Two-layer feedforward network model depicting the inputs, targets/outputs, and number of neurons for each model. Weather inputs: (i) degree days from September to harvest (DD-S-H), (ii) maximum January temperature (MJT), (iii) mean maximum temperature from veraison to harvest (MeanMaxTV-H), and (iv) mean minimum temperature from veraison to harvest (MeanMinTV-H). Sensory descriptors are found in Table 2.

Three artificial neural network (ANN) regression models were developed using a Matlab^®^ R2020a (Mathworks, Inc., Natick, MA, USA) code. A total of 17 different training algorithms were tested and compared (data not shown) to find the best models according to their performance, accuracy, and absence of overfitting signs. For Model 1, the raw absorbance values of 100 wavelengths within the 1596–2396 nm spectrum measured using the NIR device were used as inputs, while Model 2 was developed using the weather and water balance data mentioned in Section 2.2, both to predict the 19 sensory descriptors shown in Table 2. These models were constructed using the Levenberg Marquardt training algorithm with data divided randomly as 70% of samples used for training, 15% for validation with performance based on means squared error (MSE), and 15% for testing using a default derivative function (Figure 1). A neuron trimming exercise (Neurons: 3, 5, and 10) was conducted to find the best performance and no signs of overfitting.

Model 3 was developed using the weather and water balance data mentioned in Section 2.2 to predict color in three color scales (i) CIELab, (ii) RGB, and (iii) CMYK. The model was built using a random data division, with 70% of the samples used for training using the Bayesian Regularization algorithm and 30% for testing using an MSE performance algorithm (Figure 1). A neuron trimming exercise (Neurons: 3, 5, and 10) was conducted to find the model with the best performance and no overfitting signs.

## 3. Results

### 3.1. ANOVA Results

Table 3 shows the results of the ANOVA for color parameters in the three scales (CIELab, RGB, and CMYK). Significant differences were found among samples for all color parameters. It can be observed that the wine from 2014 (W14) was the darkest in color (L = 32.05) and significantly different from the other vintages, while W11 was the lightest (L = 59.23). According to the CIELab scale, W14 was the highest in “a” value, which represents the red color on the positive values, while W11 was the lowest; similarly, the R-value of W14 was the lowest (darker red), while W11 was the highest (lighter red). W08 was the highest in “b” and second lowest in G, which represent darker green colors.

**Figure 2:**
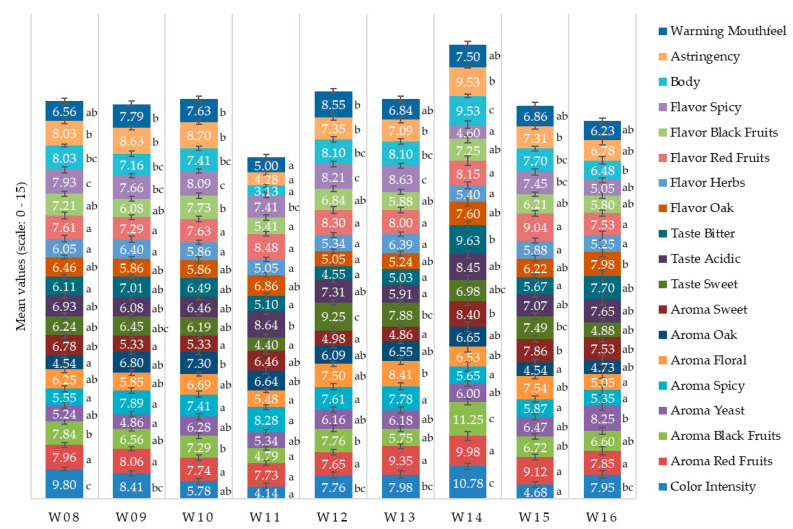
Mean values of sensory descriptors of wines from all vintages. Different letters (a–g) denote significant differences between samples based on ANOVA and Fisher’s least significant difference (LSD) *post hoc* test at α = 0.05. Sample abbreviations are described in Table 1. Error bars = standard error (range: 0.32–1.82).

Figure 2 shows the ANOVA results for the sensory descriptors. Significant differences were found among samples for all descriptors. Sample W14 presented the highest intensity for descriptors such as color, red and black fruits aroma, sweet aroma, bitter taste, body, and astringency. At the same time, it had the lowest intensity in spicy flavor. On the other hand, W11 had the lowest intensity for color, black fruits aroma, sweet taste, herbs flavor, black fruits aroma, body, astringency, and warming mouthfeel. In contrast, it had the highest intensity for spicy aroma and acidic taste. The strongest warming mouthfeel, sweet taste, and bitterness were found in W12.

### 3.2. Machine Learning Models

**Figure 3:**
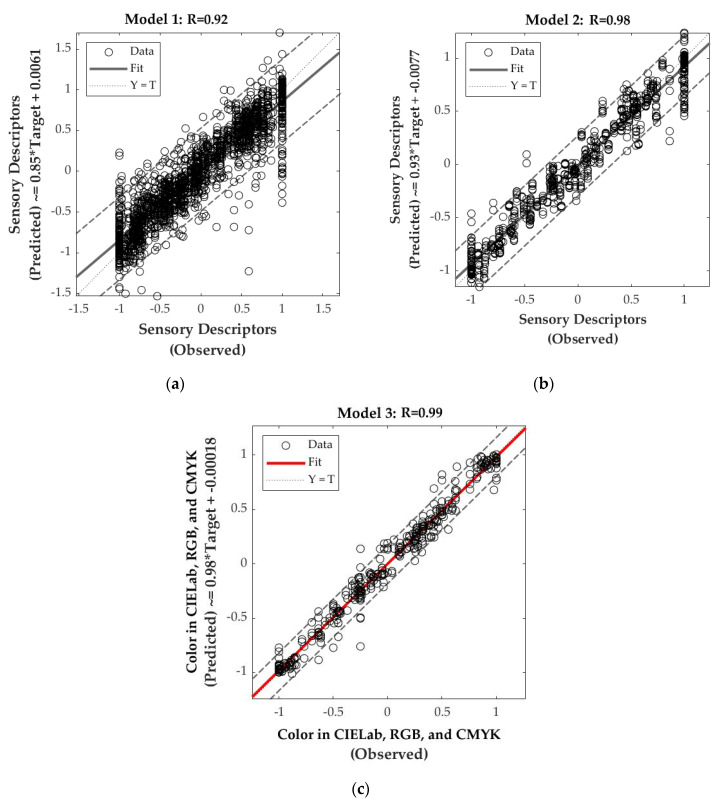
Graphs of the overall correlations for (**a**) Model 1 using near-infrared absorbance values used as inputs to predict sensory descriptors (Table 2), (**b**) Model 2 using weather information: (i) degree days from September to harvest (DD-S-H), (ii) maximum January temperature (MJT), (iii) mean maximum temperature from veraison to harvest (MeanMaxTV-H), and (iv) mean minimum temperature from veraison to harvest (MeanMinTV-H) plus (v) water balance as inputs to predict sensory descriptors (Table 2), and (**c**) Model 3 using weather and water balance data as inputs to predict color parameters in three scales (i) CIELab, (ii) RGB, and (iii) CMYK.

Table 4 shows the statistical data of the three models. It can be observed that Model 1, which was developed using NIR values as inputs to predict the intensity of sensory descriptors, presented a high overall correlation coefficient (R = 0.92; Figure 3a). However, the validation R-value (R = 0.82) is far from the training (R = 0.96), and the performance of validation (MSE = 0.68) and testing (MSE = 0.83) were not as close, which are signs of possible overfitting. Furthermore, the slope for validation is low–moderate, and the overall model presented 5.48% of outliers (103 out of 1881), based on the 95% confidence bounds (Figure 3a). In contrast, Model 2, which was developed using weather values as inputs to predict the intensity of sensory descriptors, had very high overall correlation (R = 0.98; Figure 3b) and no signs of overfitting as the validation and training R values were close (R = 0.99 and R = 0.96), and validation and testing performances are the same. Slopes from the three stages are high (slope = 0.85–0.96); the overall model presented 2.87% of outliers (36 out of 1254), based on the 95% confidence bounds (Figure 3b). On the other hand, Model 3, which was constructed using weather data as inputs to predict color parameters, had a very high overall correlation (R = 0.99; Figure 3c) and the lower training performance (MSE < 0.01) compared to the testing (MSE = 0.02), shows that there were no signs of overfitting. Furthermore, slope values were high and close to unity (slope ~1), while the overall model presented 3.33% of outliers (22 out of 660), according to the 95% confidence bounds (Figure 3c).

## 4. Discussion

Weather information for contrasting seasons for the same vineyard has been previously reported [9,50]. From all nine seasons, the most contrasting vintage was 2011, presenting higher and anomalous rainfall with lower irrigation input, resulting in a water balance of 673.7 mm and lowest solar exposure between veraison and harvest of 15.6 MJ m^−2^. Higher water availability will increase canopy vigor and offset canopy balance towards the vegetative fraction over reproductive (grapes). This explains lower color (Table 3) and sensory profiles of wines that resulted from this particular vintage (Figure 2), consistent with previous studies [60,61]. On the contrary, the 2013 and 2014 vintages were related to lower water balance (−117.5 and −61.9 mm respectively) and higher solar exposure between veraison to harvest (21.8 and 19.0 MJm^−2^, respectively) with warmer temperatures. These vintages produced wines with the highest color (Table 3) and sensory quality traits (Figure 2). Color is an important quality trait for Pinot Noir wines, and its prediction before winemaking can offer powerful decision-making tools to winegrowers [62,63].

The use of the CIELab color scale in food and beverages is attributed to its uniform distribution of color in the scale and considered as the closest to the human eye perception of colors. However, RGB has also been reported to be similar to human perception [64] and has been used in food studies such as oil, beer, and wine [65,66,67]. The latter scale has been found to be correlated with pigments such as carotenoids in olive oil [66] and used to predict adulteration in wines [67]. On the other hand, despite that CMYK is not utilized in food, it may provide useful information to print the corresponding color on labels to increase consumer perception before opening the bottle. According to Piqueras-Fiszman et al. [68,69], it is very important for packaging to display the real colors of the contained product to ease consumer familiarization with the food or beverage. Furthermore, Lick et al. [70] found that there is an association between the colors in labels and the flavors that consumers expect in the wine.

Within the 1596–2396 nm NIR range, overtones of several components may be found. Some of these compounds that are related to the sensory descriptors are aromatics (1685 nm), water (1790 and 1940 nm), carboxylic acids, which form esters that are common aromatic compounds (1900 nm), pOH that is related to acidity and inverse scale to pH (1908 nm), alcohol (2090 nm), sucrose (2080 nm), and citric acid (2220 nm), among others. Furthermore, intensities of basic tastes rated by a trained panel have been modeled to be predicted using NIR absorbance values within the aforementioned range in chocolate, which indicates there is an association between this wavelength range and sensory attributes [71].

Machine learning modeling has been previously implemented to predict aroma profiles for the same vintages reported in this study, and aroma patterns are consistent with the sensory results presented here (Figure 2) [9]. Aroma profiles are also dependent on canopy architecture and the vegetative and reproductive balance, similar to other crops, such as cocoa trees, which have also been modeled using machine learning [72]. These modeling techniques have been proven to be accurate and robust to predict aroma and sensory profiles of other beverages as per recent research published on artificial intelligence, robotics, computer vision, and machine learning applications to beverages [73,74,75].

The ML model based on chemical fingerprinting of wines using NIR (Model 1) was not as accurate compared to Models 2 and 3 based on weather and management information from vertical vintages. Further disadvantages of Model 1 are related to the requirement of the NIR instrument, which can be cost-prohibitive to winegrowers and winemakers, and measurements are obtained after winemaking. However, it could offer a quick assessment of wines produced without the requirement of trained sensory panels, which in turn can be time-consuming and cost-prohibitive and not accessible for most wineries. The implementation of Model 1 could offer a rapid, robust, accurate, and reproducible way to assess the sensory profile of wines and wine batches to maintain a certain wine style that characterizes specific wineries.

More practical and accurate models developed in this study were based on weather information and water management of vineyards (Models 2 and 3) to predict sensory profiles and color of the wines, respectively. The effect of seasonal variability on soil, grapevine, environment, and water management, and its influence on the quality traits in grapes and wines have been well-established. Models 2 and 3 offer information on sensory profiles and wine traits before harvest and winemaking. These models will offer the opportunity to winemakers to adjust vinification techniques to obtain a more consistent wine style, predict the market and consumer acceptance for pricing adjustments, better description of wines in labels for accurate information to consumers, among others.

Models 1 to 3 are specific to the location and corresponding wine and winemaking techniques; thus, they could have very limited applicability for other vineyards, wineries, and wines from different soil types, climatic regions, and cultivars. However, the methodology is very easy to reproduce to obtain specific models when libraries of vertical wines and meteorological information are available through the years. Furthermore, once the models are constructed per winery, region, and cultivars, weather information projections can be incorporated for early prediction of sensory profile and color of resulting wines. Even though the models can be considered as site-specific and variety specific, by adding more data, they have the capability to “learn”, hence making them more broadly applicable to other environments and cultivars.

Temperatures and rainfall, which were the basis of weather parameters in this paper, can be obtained for up to three months in advance for any specific region in Australia from the Bureau of Meteorology (BOM, Outlook information, Australia). From this information, evapotranspiration (ET) and water balance data can be estimated early in the season by applying ET predictive models based on temperature [76,77] and corresponding Kc values. Earlier prediction (three months in advance) will be associated with higher estimation errors of temperature and ET and overall outputs for Model 1 and 2. However, periodic model feeding from veraison onwards will offer reference information for changes of sensory and color trends for wines, which may be used as a decision-making tool to schedule irrigation and canopy management within the season.

One of the main disadvantages found through this research was related to putting all the historical information together from vineyards. It is common that these industries have a mix of information and data recorded manually (handwritten), and printed but not recorded digitally, based on different software platforms (i.e., Excel, Word, database platforms) or specific database commercial software. Furthermore, it could be considered as a disadvantage the specialized analysis required to construct the models proposed here concerning the physicochemical and sensory analysis of vertical libraries of wines available. Recent studies and developments have made it possible to implement new and emerging technologies to make these analyses more affordable and user-friendly. Some of these are, for example, the development of robotic pourers coupled with computer vision, machine learning and gas release analysis of beers [65,78] and sparkling wines [75], low-cost electronic noses for aroma profile and faults detection [73], low-cost near-infrared spectroscopy devices and color sensors that can be attached to smartphones with applications in food and beverages [50,79], and sensory analysis of consumers using a newly developed computer application, which can be downloaded by users and deployed in Android-based devices to obtain normal sensory analysis (self-reported) plus biometrics for emotional response and physiological changes of participants, such as heart rate, blood pressure [80], and body temperature among others [59].

## 5. Conclusions

This study is one of the first attempts to apply these techniques for the assessment of vertical vintages in the wine industry, which have offered encouraging results with the construction of robust machine learning models with high accuracy and practicality. Models presented in this study were based on new and emerging technologies (near-infrared spectroscopy) and ubiquitous weather information from past seasons and relevant vineyard management data applied to the vertical library of wines that are mostly available in the majority of vineyards around the world. Further research should be conducted to incorporate more cultivars, seasonality, and winemaking techniques to create more robust machine learning models to assess final wine aroma profiles, sensory profiles, and color. This research is the first step to achieve universal machine learning models to apply artificial intelligence to the winemaking industry. These models and procedures may be considered preliminary. However, they have the following advantages: (i) easy to construct site-specific models for other regions and cultivars using vertical vintages and historical meteorological data, (ii) models can incorporate future seasons and use the intrinsic “learning” capabilities of these methodologies to incorporate climate change factors that may affect targets proposed, (iii) models were constructed based on information that can be considered nowadays ubiquitous and wineries that keep vintage libraries of wines can get full benefits from these procedures. The main disadvantage of obtaining these benefits could be related to the physicochemical and sensory analysis of wines required to construct the models. However, recently, there has been a body of research to make these measurements more affordable and accessible to the industries in a “do-it-yourself” fashion.

## Figures and Tables

**Table 1:** Sample vintages used for the study, including labels/abbreviations, alcohol content, and pH.

Wine Vintage	Label/Abbreviation	Alcohol Content	pH
2008	W08	13.7%	3.7
2009	W09	13.9%	3.6
2010	W10	13.9%	3.7
2011	W11	13.7%	3.6
2012	W12	14.2%	3.6
2013	W13	13.6%	3.6
2014	W14	13.6%	3.8
2015	W15	14.2%	3.7
2016	W16	13.7%	3.5

**Table 2:** Descriptors evaluated in the sensory session, and the anchors used in the line-scale.

Descriptor	Anchors
Color intensity	Light–Dark
Red fruits aroma	Absent–Intense
Black fruits aroma	Absent–Intense
Yeast aroma	Absent–Intense
Spicy aroma	Absent–Intense
Floral aroma	Absent–Intense
Oak aroma	Absent–Intense
Sweet aroma	Absent–Intense
Sweet taste	Absent–Intense
Acidic taste	Absent–Intense
Bitter taste	Absent–Intense
Oak flavor	Absent–Intense
Herbs flavor	Absent–Intense
Red fruits flavor	Absent–Intense
Black fruits flavor	Absent–Intense
Spicy flavor	Absent–Intense
Body	Light–Full
Astringency	Absent–Intense
Warming mouthfeel	Absent–Intense

**Table 3:** Mean values of color parameters of wines from all vintages. Different letters (a–g) denote significant differences between samples based on ANOVA and Fisher’s least significant difference (LSD) *post hoc* test at α = 0.05.

Sample	L	SE	a	SE	b	SE	R	SE	G	SE	B	SE	C	SE	M	SE	Y	SE	K	SE
W08	38.35b	0.60	31.98e	0.45	20.57f	0.20	144.33b	2.03	67.33b	1.20	59.00a	1.53	0.30b	0.01	0.80g	<0.01	0.75f	0.01	0.25d	0.01
W09	44.38c	0.67	28.71cd	0.30	17.29e	0.06	155.83c	2.13	85.00c	1.32	78.17b	1.76	0.30b	0.01	0.72e	<0.01	0.65e	0.01	0.18c	0.01
W10	50.41e	0.79	25.45b	0.17	14.01d	0.17	167.33d	2.33	102.67de	1.67	97.33cd	2.33	0.30b	0.01	0.65c	0.01	0.56d	0.01	0.10b	0.01
W11	59.23g	0.62	18.11a	0.09	12.17c	0.29	180.33e	1.45	130.67g	1.76	122.33f	1.86	0.29ab	<0.01	0.51a	0.01	0.47b	0.01	0.03a	0.01
W12	51.98e	0.38	26.44b	0.46	13.31cd	0.47	173.00d	0.58	106.00e	1.16	102.33de	1.45	0.29ab	<0.01	0.65c	<0.01	0.53c	0.01	0.08b	<0.01
W13	50.40e	0.73	29.65d	0.74	14.43d	0.88	173.33d	2.91	99.00d	1.52	97.00c	2.08	0.28a	0.01	0.68d	0.01	0.56d	0.02	0.09b	0.01
W14	32.05a	0.69	37.13g	1.26	12.46c	0.47	131.00a	3.22	46.67a	1.45	58.00a	1.53	0.33c	0.01	0.89h	0.01	0.68e	0.01	0.32e	0.02
W15	55.55f	0.57	27.06bc	0.50	7.03b	0.21	181.00e	2.41	115.00f	1.02	121.89f	1.31	0.28a	0.01	0.62b	<0.01	0.42a	0.01	0.03a	<0.01
W16	47.97d	0.68	35.11f	0.67	5.63a	0.39	170.33d	2.67	89.00c	1.53	105.67e	1.76	0.30b	0.01	0.75f	0.01	0.46b	0.01	0.07b	0.01

Abbreviations: SE- Standard error; L, a, and b -parameters from CIELab scale; R, G and B -Red, Green, and Blue from RGB scale; and C, M, Y, K - Cyan, Magenta, Yellow and Black from CMYK color scale. Sample abbreviations are described in Table 1.

**Table 4:** Statistical results from the three artificial neural network models.

Stage	Samples	Observations (Samples × Targets)	R	Performance (MSE)	Slope
**Model 1 (Near-infrared inputs; Sensory targets)**					
Training	69	1311	0.96	0.03	0.90
Validation	15	285	0.82	0.16	0.68
Testing	15	285	0.82	0.13	0.83
Overall	99	1881	0.92	-	0.85
**Model 2 (Weather inputs; Sensory targets)**					
Training	46	874	0.98	0.01	0.96
Validation	10	190	0.96	0.04	0.85
Testing	10	190	0.96	0.04	0.85
Overall	66	1254	0.98	-	0.93
**Model 3 (Weather inputs; Color targets)**					
Training	46	460	0.99	<0.01	0.98
Testing	20	200	0.97	0.02	0.98
Overall	66	660	0.99	-	0.98

Abbreviations: R = correlation coefficient, MSE = means squared error.
